# Editorial: Plasticity of Endogenous Pain Modulatory Circuits in Neuropathy

**DOI:** 10.3389/fpain.2021.776948

**Published:** 2021-10-21

**Authors:** Ryan Patel, Bridget M. Lumb, Kirsty Bannister

**Affiliations:** ^1^Wolfson Centre for Age Related Diseases, King's College London, London, United Kingdom; ^2^School of Physiology, Pharmacology and Neuroscience, University of Bristol, Bristol, United Kingdom

**Keywords:** neuropathic pain, endogenous pain modulation, descending pain control, animal models, fMRI, chronic pain, monoamines, endocannabinoids

## Background

Acute pain has a physiological role to protect against tissue damage, however chronic pain can persist as a sequela of a multitude of diseases due to maladaptive plasticity in the peripheral and central nervous system. New treatment algorithms are desperately needed as it is clear that when treated as a homogenous patient population many fail to manage their pain effectively (1, 2).

Early seminal studies demonstrated that micro-stimulation of the mid-brain produced a profound analgesia (3). It was later shown that this modulation is bi-directional and serves to amplify or suppress spinal sensory transmission. At rest these descending controls are balanced to fine-tune sensory gain but can rapidly adapt depending on context, expectation, and emotional state. Key examples include placebo analgesia, offset analgesia/onset hyperalgesia, attentional analgesia, stress-induced analgesia/hyperalgesia, and conditioned pain modulation. Each of these co-exist within the descending pain modulatory system (DPMS) and depend on partially overlapping signalling systems and circuits. For example, placebo analgesia in part requires descending opioidergic pathways (4), conditioned pain modulation is mediated by mainly noradrenergic and partly opioidergic signalling (5, 6), whereas attentional analgesia, stress-induced analgesia/hyperalgesia and offset analgesia are associated with the periaqueductal grey-dorsal raphe axis (7–9).

There is an extensive body of pre-clinical evidence that links altered descending control to the development and maintenance of neuropathic pain states (10–13), and these findings are supported by clinical studies based on imaging or psychophysical tests. The most widely studied in patients is conditioned pain modulation (CPM), also referred to as diffuse noxious inhibitory controls, which may represent a translatable endpoint linking pre-clinical and clinical investigations (14). This form of dynamic quantitative sensory testing is often simplified as “pain inhibits pain” where two distant noxious stimuli produce analgesia, and likely represents a surrogate measure of the net balance of descending facilitatory and inhibitory signalling. Inefficient CPM provides an insight into pathophysiological mechanisms and has been observed in neuropathic and chronic pain conditions (6, 15–17). Inefficient CPM has also been linked to “pain vulnerability” for post-operative pain (18, 19), and could be useful as a sensory biomarker for mechanism-led treatment selection as tapentadol/duloxetine efficacy are inversely correlated with CPM efficiency in neuropathic patients (6, 20).

Clearly there is still much to be learned about the mechanisms and circuits that subserve different aspects of the DPMS. This may ultimately help define individual differences in pain mechanisms, pain vulnerability, and guide treatment selection. Within this Research Topic we collated original research and review articles providing novel insight into the function of the DPMS in neuropathic pain.

## Overview of the Articles Included in This Research Topic

In their original research article Osborne et al. perform a longitudinal study to examine differences in subgenual anterior cingulate cortex (sgACC) functional connectivity in patients with carpel tunnel syndrome and those that recovered after surgery. The sgACC is thought to engage structures within the DPMS such as the periaqueductal grey, dorsal raphe, and prefrontal cortex implicating a role in endogenous pain modulation. In healthy individuals, sex differences have been observed in resting functional connectivity; women had greater connectivity with the DPMS pathways (periaqueductal grey and raphe nuclei) whereas men showed greater engagement of salience networks. In carpel tunnel syndrome, sgACC functional connectivity with the prefrontal cortex and temporal lobes was found to be reduced in men but not women when compared to healthy controls. In addition to sex differences in the dynamic connectome, plasticity in sgACC functional connectivity was observed following surgical treatment. Prior to surgery, patients had stronger sgACC functional connectivity with the orbitofrontal cortex, dorsal striatum, premotor cortex, and middle frontal gyrus compared with after surgery. After surgery patients had greater sgACC connectivity with the posterior insula and central/parietal operculum compared with their pre-operative scans.

In their original research article Boullon et al., studied sex differences in the development of neuropathic pain in a rat model of traumatic nerve injury and associated levels of endocannabinoids. Female rats exhibited earlier onset of hypersensitivity and increased sensitivity to cold and mechanical stimulation. Co-morbidities were also examined though neither sex exhibited neuropathy-induced changes in locomotion, anxiety-, hedonistic-, or depression-like behaviours. Endocannabinoids are widely expressed throughout the DPMS and have an established role in modulating nociceptive transmission. Endocannabinoid levels were measured in key nodes of the DPMS including the prefrontal cortex, periaqueductal grey, rostral ventromedial medulla, and amygdala though no injury dependent changes in endocannabinoid levels were observed at the time-point tested.

In their review article Tavares et al. discuss plasticity of monoaminergic and opioidergic brainstem pathways in pre-clinical models of pain. Of particular focus is the facilitatory role of the dorsal reticular nucleus, the bi-directional function of the dorsal raphe nuclei, and the inhibitory function of the locus coeruleus. In addition they provide an overview of the opioidergic modulation of these regions. Lastly they discuss the alterations in these signalling systems in models of neuropathic pain and the implications for treatment of patients.

Lastly, in their review article Mills et al. discuss how fMRI studies have provided insight into DPMS function. The main focus is functional connectivity between brainstem nuclei and the spinal cord, and how activity in these networks are disrupted in neuropathy. In addition they discuss the mechanisms that may underlie conditioned pain modulation, and differences in functional connectivity in subjects with and without effective conditioned pain modulation.

## Author Contributions

All authors listed have made a substantial, direct and intellectual contribution to the work, and approved it for publication.

## Conflict of Interest

The authors declare that the research was conducted in the absence of any commercial or financial relationships that could be construed as a potential conflict of interest.

## Publisher's Note

All claims expressed in this article are solely those of the authors and do not necessarily represent those of their affiliated organizations, or those of the publisher, the editors and the reviewers. Any product that may be evaluated in this article, or claim that may be made by its manufacturer, is not guaranteed or endorsed by the publisher.
